# The Kinetics of SARS-CoV-2 Antibody Development Is Associated with Clearance of RNAemia

**DOI:** 10.1128/mbio.01577-22

**Published:** 2022-06-28

**Authors:** Chuangqi Wang, Yijia Li, Paulina Kaplonek, Matteo Gentili, Stephanie Fischinger, Kathryn A. Bowman, Moshe Sade-Feldman, Kyle R. Kays, James Regan, James P. Flynn, Marcia B. Goldberg, Nir Hacohen, Michael R. Filbin, Douglas A. Lauffenburger, Galit Alter, Jonathan Z. Li

**Affiliations:** a Department of Biological Engineering, Massachusetts Institute of Technology, Cambridge, Massachusetts, USA; b Brigham and Women’s Hospital, Boston, Massachusetts, USA; c Massachusetts General Hospital, Boston, Massachusetts, USA; d Ragon Institute of MGH, MIT and Harvard, Cambridge, Massachusetts, USA; e Broad Institute of MIT and Harvard, Cambridge, Massachusetts, USA; f Department of Microbiology, Harvard Medical School, Boston, Massachusetts, USA; Johns Hopkins Bloomberg School of Public Health

**Keywords:** longitudinal data modeling, persistent SARS-CoV-2 plasma viremia, system serology, viremia, humoral immune response

## Abstract

Persistent SARS-CoV-2 replication and systemic dissemination are linked to increased COVID-19 disease severity and mortality. However, the precise immune profiles that track with enhanced viral clearance, particularly from systemic RNAemia, remain incompletely defined. To define whether antibody characteristics, specificities, or functions that emerge during natural infection are linked to accelerated containment of viral replication, we examined the relationship of SARS-CoV-2-specific humoral immune evolution in the setting of SARS-CoV-2 plasma RNAemia, which is tightly associated with disease severity and death. On presentation to the emergency department, S-specific IgG3, IgA1, and Fc-γ-receptor (Fcγ R) binding antibodies were all inversely associated with higher baseline plasma RNAemia. Importantly, the rapid development of spike (S) and its subunit (S1/S2/receptor binding domain)-specific IgG, especially FcγR binding activity, were associated with clearance of RNAemia. These results point to a potentially critical and direct role for SARS-CoV-2-specific humoral immune clearance on viral dissemination, persistence, and disease outcome, providing novel insights for the development of more effective therapeutics to resolve COVID-19.

## INTRODUCTION

Accumulating evidence has shown that SARS-CoV-2 systemic dissemination, as manifested by levels of plasma RNAemia, is linked to more extensive tissue damage, endothelial inflammation, and coagulopathies and predicts the risk of eventual disease severity and death (1–3). In immunosuppressed individuals, persistent SARS-CoV-2 RNAemia has also been detected and is thought to play a role in disease pathogenesis (4) and can lead to additional selection and the outgrowth of viral variants (5). Thus, the development of therapeutics able to rapidly control and contain SARS-CoV-2 viral replication is key not only for the management of COVID-19 disease but also in preventing postinfection complications and the evolution of novel viral variants. The recent development of novel therapeutics able to reduce the risk of severe COVID-19 has effectively revolutionized our ability to fight against SARS-CoV-2; however, new small molecules still only provide partial effectiveness in those with most severe disease (6). Instead, both convalescent plasma and monoclonal therapeutics were deployed throughout the pandemic with the hope that these virus-specific therapeutics could effectively reverse the trajectory of disease. While convalescent plasma resulted in mixed results, monoclonal therapeutics displayed therapeutic benefit in early infection but poor benefit in late and severe COVID-19 (7, 8). However, monoclonal therapeutics were largely optimized for neutralizing antibody potency but did not fully explore the full functional potential of these unique therapeutics. However, whether additional antibody functions, beyond neutralization, are linked to enhanced clearance and control of the virus, particularly in the plasma, remains unclear.

There have been conflicting data on the role of the humoral immune response in resolution of COVID-19, with some studies reporting no significant association between viral load and anti-SARS-CoV-2 antibody responses, some studies pointing to higher antibody levels present in severe disease, and still other studies showing that monoclonal antibody therapy and host antibody development can reduce and control viral dynamics (9–11). However, many of these studies analyzed the effect of antibody development on upper respiratory tract viral dynamics, but respiratory tract viral loads do not always reflect levels of plasma RNAemia (3), the latter linked more closely to disease outcomes (1, 3). In addition, the longitudinal kinetics of antibody development, rather than a snapshot of antibody levels at a certain disease point, may be key to defining mechanisms of differential control of viral dissemination, as has been observed for predicting disease outcome (12). Moreover, studies have focused largely on binding and neutralizing activity, rather than the broader role of antibodies on leveraging the immune system to clear the virus and infected cells. Thus, it remains unclear whether the evolution of specific antibody functions or qualities may be key to antiviral control outside the respiratory tract. The insights gained from addressing this question could shed light upon the development of next-generation monoclonal therapeutics with expanded therapeutic capacity to prevent disease progression later in the course of infection. To this end, we sought to holistically profile the evolution of the SARS-CoV-2 humoral response with respect to the dynamics of RNAemia in a cohort of COVID-19 patients presenting just days after symptom onset.

## RESULTS

### Lower IgG3 and lower FcγR binding levels track with higher RNAemia at the time of emergency department (ED) presentation.

While the kinetics of the evolution of the SARS-CoV-2-specific humoral immune response (12) and plasma RNAemia (1, 3) have both been associated with disease severity, it remains unclear whether SARS-CoV-2 antibodies track directly with differential systemic viral control. Using a cohort of acutely ill COVID-19 individuals, the relationship and kinetics of the humoral immune response were investigated. A total of 300 participants presenting to the emergency department (ED) with respiratory distress were included in this study, with 53 participants harboring RNAemia above the quantification range (2 log_10_ copies/mL) and 53 with detectable RNAemia below the level of quantification (1.6 log_10_ copies/mL) (1, 13). Approximately 40% of participants were at least 65 years old, 48% were female, 12% obese, 36% diabetic, and 48% hypertensive. In addition, 13% of participants had kidney disease, 21% had lung disease, 15% had heart disease, and 8% were immunocompromised. The median duration of symptoms before presentation was 7 days.

**FIG 1:**
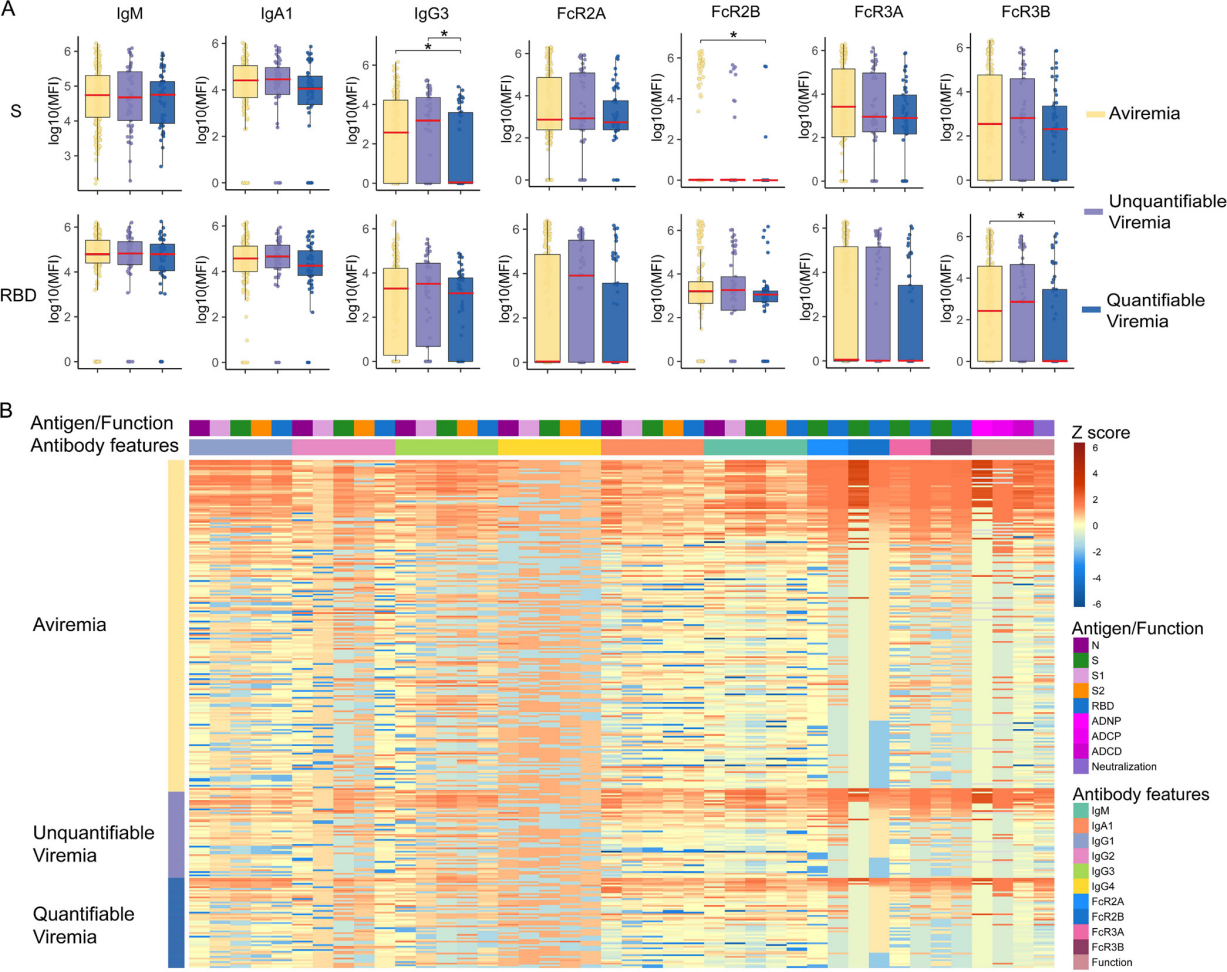
Baseline antibody levels by levels of viremia. (A) Baseline S- and RBD-specific antibody features among different viremia groups (*n* = 300). Three viremia groups’ levels were compared using Mann-Whitney U tests with Benjamini-Hochberg correction (*, *P* < 0.05). Tukey boxplots were used to demonstrate the antibody levels and functions, with boxes indicating medians (red line) and interquartile ranges. Upper whiskers indicate the 75th percentile value plus 1.5 · interquartile range, and lower whiskers indicate the 25th percentile value minus 1.5 · interquartile range. (B) Heatmap summarizing baseline antibody features among three viremic groups.

System serology was utilized to profile the evolution of the SARS-CoV-2-specific humoral immune response, capturing the evolution of distinct antibody isotypes and subclasses, IgG Fcγ-receptor binding profiles, neutralization levels, and Fc-effector functions (monocyte phagocytosis, neutrophil phagocytosis, and complement deposition). For baseline analysis, all 300 participants were included. As shown in Fig. 1A, all the participants already had a detectable spike (S)-IgM antibody response. Elevated plasma RNAemia at the time of ED presentation was associated with lower S-specific IgG3 levels. IgG Fc-receptor spike receptor binding domain (RBD)-specific Fcγ R3B and S-specific Fcγ R2B binding were elevated in aviremic individuals compared to subjects with quantifiable RNAemia (Fig. 1A). Conversely, there were no significant differences in baseline antibody-dependent neutrophil phagocytosis (ADNP), antibody-dependent cellular phagocytosis (ADCP), and antibody-dependent complement deposition (ADCD) among different RNAemia groups (Fig. 1B; see Fig. S1 in the supplemental material).

10.1128/mbio.01577-22.1 FIG S1 Baseline antibody levels by levels of viremia (related to Fig. 1). Baseline N, S1, S2, S, and RBD-specific antibody features among different viremia groups (*n* = 300). Three viremia groups’ levels were compared using Mann-Whitney *U* tests with Benjamini-Hochberg correction (*, *P* < 0.05). Tukey boxplots demonstrated the antibody levels and functions, with boxes indicating medians (red line) and interquartile ranges. Upper whiskers indicate 75th percentile value plus 1.5 · interquartile range, and lower whiskers indicate 25th percentile value minus 1.5 · interquartile range. (A) Antibody titer levels. (B) FcγR binding levels. (C) Antibody-dependent neutrophil phagocytosis (ADNP), antibody-dependent cellular phagocytosis (ADCP), antibody-dependent complement deposition (ADCD), and neutralization levels. Download FIG S1, TIF file, 1.5 MB. Copyright © 2022 Wang et al. 2022 Wang et al. https://creativecommons.org/licenses/by/4.0/ This content is distributed under the terms of the Creative Commons Attribution 4.0 International license.

### Persistence of plasma RNAemia is associated with increased COVID-19 mortality.

**FIG 2:**
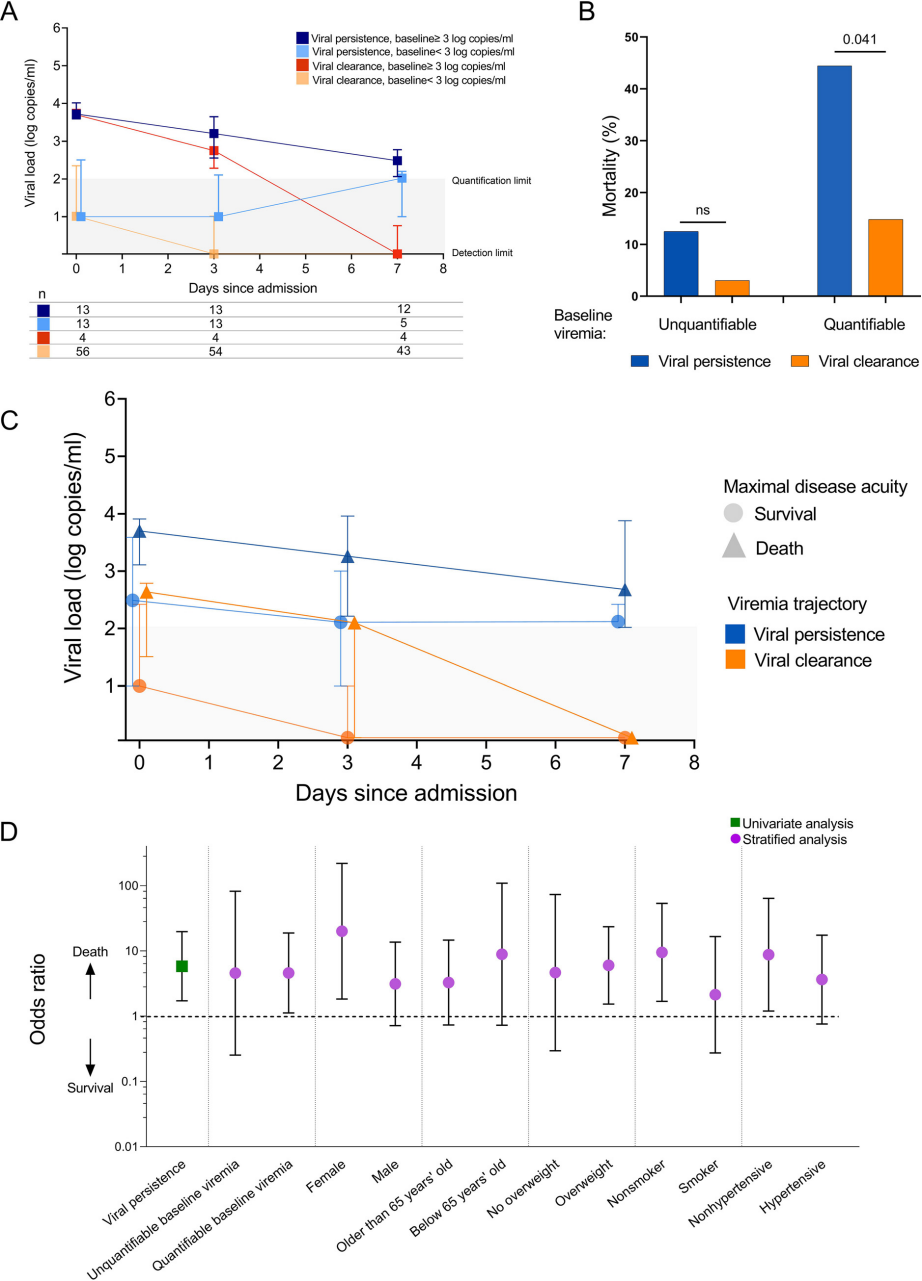
Longitudinal trajectory of SARS-CoV-2 viremia. (A) Viremia trajectory stratified by baseline viremia level and viremia clearance (*n* = 86). Medians and interquartile ranges are shown. (B) Viremia persistence is associated with mortality. Fisher’s exact test was used to evaluate the statistical differences between the persistence and clearance groups. (C) Viremia trajectory stratified by COVID-19 disease outcome. Medians and interquartile ranges are shown. (D) Odds ratio of death in those with viral persistence in a univariate analysis (green square) and in logistic regression (purple circles) stratified by baseline viral load, demographics, and other potential mediators of increased mortality. Odds ratios with 95% confidence intervals were demonstrated.

The essential role of antibodies in the elimination of viruses has been shown for pulmonary infections such as influenza (14), respiratory syncytial virus (15), and systemic pathogens including Ebola virus (16) and, most recently, malaria (17). To first determine whether viral clearance was associated with COVID-19 severity, we analyzed viral clearance dynamics in 86 viremic participants who were hospitalized and had more than one follow-up time point, permitting longitudinal analysis. While RNAemia trended down over time, a subset of participants did not fully clear their RNAemia within 7 days of hospitalization (Fig. 2A). These individuals were grouped in the “viral persistence” category in contrast to individuals that cleared the virus at the second time point, also referred to as the “viral clearance” category.

The viral persistence and viral clearance groups had comparable duration of symptoms prior to ED presentation (seven versus 8 days, *P = *0.30), and the majority of participants presented to the ED within 15 days of symptom onset (Fig. S2A). Similar to our previous analyses in hospitalized patients (1, 3), baseline quantifiable plasma RNAemia at the time of ED presentation was associated with an increased mortality risk. Importantly, the presence of persistent RNAemia was independently associated with risk of death. Individuals with both quantifiable RNAemia at baseline and viral persistence had the greatest risk of death (44%, Fig. 2B), while those with unquantifiable baseline RNAemia and no evidence of viral persistence had the lowest risk of death (3%). The trajectory of RNAemia was further stratified by disease severity, and in both the viral clearance and persistence groups, those who survived COVID-19 demonstrated lower levels of RNAemia (Fig. 2C, Fig. S2B to D). Viral persistence was associated with an odds ratio (OR) of 5.8 (95% confidence interval [CI], 1.7 to 19.7; *P = *0.005) with mortality (Fig. 2D). After stratifying for baseline RNAemia and other baseline characteristics, viral persistence was consistently associated with increased mortality across different subgroups (Fig. 2D). Baseline kidney disease and hypertension were significantly associated with the persistence of RNAemia (Fig. S2E). Using RNA levels from day 7 as a surrogate for viral clearance, we also noted a significant association between death and day 7 RNA levels (Fig. S2F).

10.1128/mbio.01577-22.2 FIG S2 Viremia dynamics during hospitalization days 0, 3, and 7. (A) Symptom duration was comparable between the persistence and clearance groups. The Mann-Whitney test was used to compare two groups. (B) Trajectory of viremia level stratified by baseline viral load in each participant. (C) Summary of viral load based on viral clearance. The Mann-Whitney test was used to compare two groups, and the Benjamini-Hochberg procedure was used to adjust for multiple comparison. (D) Trajectory of viremia level stratified by viral clearance in each participant. (E) Viral persistence was rate stratified by different comorbidities. *P* values were calculated by either Chi-squared test or Fisher exact test. (F) Death was significantly associated with higher RNA levels at day 7 (Mann-Whitney test). *, *P* < 0.05; **, *P* < 0.01; ***, *P* < 0.001. Download FIG S2, TIF file, 0.6 MB. Copyright © 2022 Wang et al. 2022 Wang et al. https://creativecommons.org/licenses/by/4.0/ This content is distributed under the terms of the Creative Commons Attribution 4.0 International license.

### Clearance of RNAemia tracks with SARS-CoV-2-specific antibody evolution.

**FIG 3:**
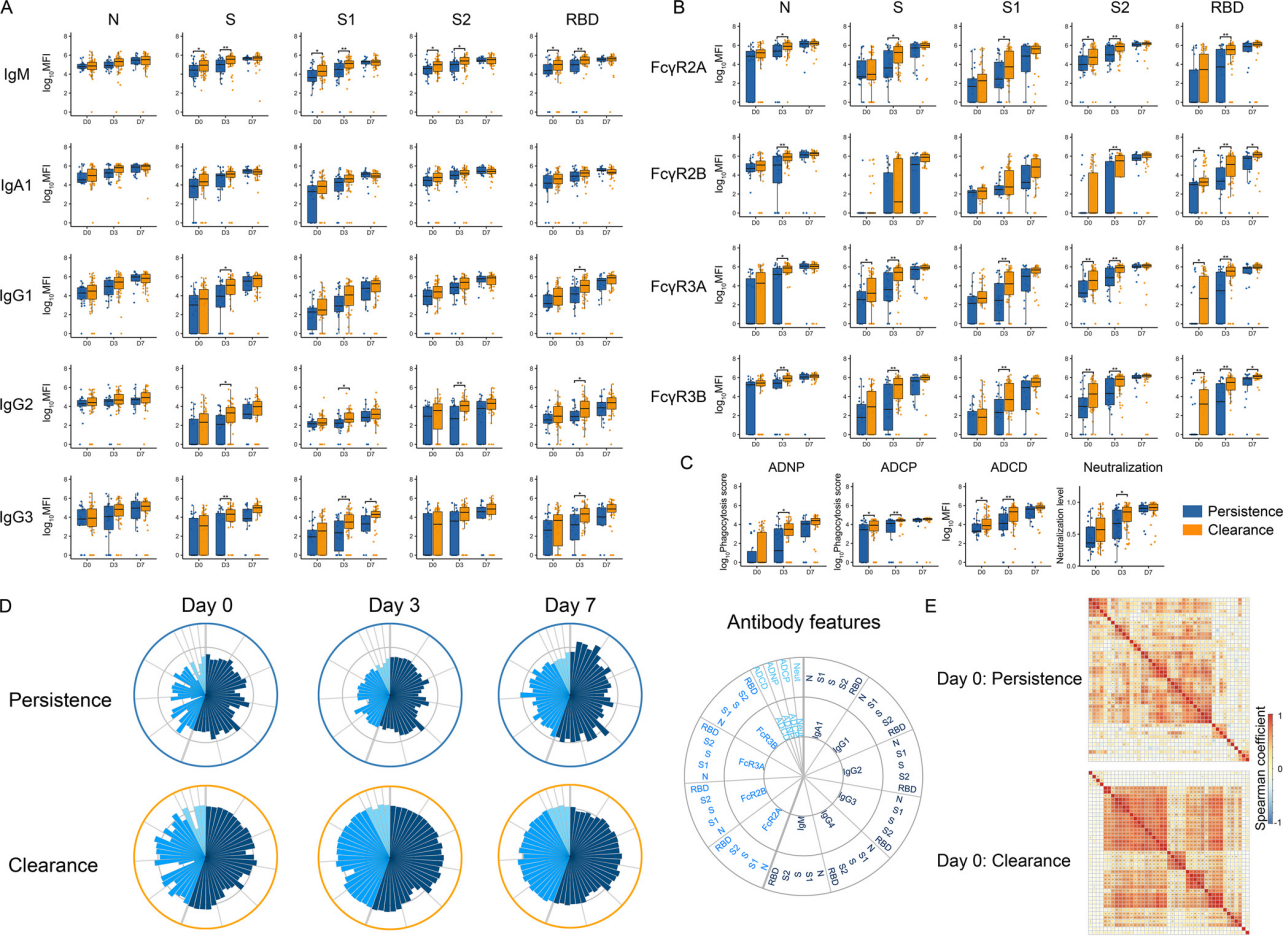
Longitudinal antibody trajectories across individuals that cleared or experienced persistent viremia. Plasma samples (*n* = 234) from 86 hospitalized SARS-CoV-2 infected individuals were profiled. (A to C) Distributions of (A) immunoglobulin titers, (B) Fc-receptor binding levels, and (C) antibody functions over admission days 0, 3, and 7 of hospitalization across viremia clearance (orange) and persistence (blue) groups. The whisker plots show the distribution, the solid black line represents the median, and the box boundary (upper and lower) represents the first and third quartiles. The dots show the scaled values of each sample. A two-sample Wilcox rank test was used to evaluate the differences between two groups for all the intervals and features. The *P* values were corrected from multiple hypothesis testing using the Benjamini-Hochberg procedure per each feature. Significance corresponds to adjusted *P* values (*, *P* < 0.05; **, *P* < 0.01). (D) The polar plots depict the mean percentile of each antibody feature at days 0, 3, and 7 across the persistence (top) and the clearance (bottom) groups. The major slices represent titer, Fc-receptors, and functions. The size of the wedge depicts the mean percentile, ranging from 0 to 0.75. (E) The Spearman correlation coefficient heatmap at day 0 across different participants who cleared viremia or experienced persistent viremia. Detailed information is shown in Fig. S3.

We next evaluated the dynamics of RNAemia and antibody evolution across the same participants based on whether they experienced viral persistence or clearance. Early antibody responses, specifically IgM responses against S/S1/S2, were significantly different across the groups, at both day 0 and day 3 (Fig. 3A). SARS-CoV-2-specific IgG 1/2/3 against S and RBD were most different between the groups at day 3. However, by day 7, the clearance and persistence groups had comparable antibody levels (Fig. 3A). In parallel to IgG1/2/3 levels, SARS-CoV-2-specific Fcγ R binding antibody levels also developed differentially across individuals who experienced viral persistence compared to those who cleared RNAemia. N-, S-, and S1/S2/RBD-specific responses all exhibited higher Fcγ R2A/3A/3B binding levels at day 3 following admission in subjects that cleared infection compared to the subjects who experienced persistent RNAemia (Fig. 3B). Importantly, significantly higher levels of FcγR3A and 3B binding related to RBD- and S2-specific antibodies were present in participants who cleared RNAemia as early as day 0 of the study.

10.1128/mbio.01577-22.3 FIG S3 Correlation among different antibody features at day 0 in the persistence and clearance groups (related to Fig. 3E). Correlation heatmaps were generated using the pairwise Spearman correlation coefficient among different antibody features across patients. (A) Persistence group. (B) Clearance group. *, *P* < 0.05, **, *P* < 0.01; ***, *P* < 0.001. Download FIG S3, TIF file, 2.9 MB. Copyright © 2022 Wang et al. 2022 Wang et al. https://creativecommons.org/licenses/by/4.0/ This content is distributed under the terms of the Creative Commons Attribution 4.0 International license.

Similar to antibody levels and FcγR binding activity, the evolution of antibody function tracked with clearance of RNAemia. Antibody-dependent complement deposition (ADCD) and antibody-dependent cellular phagocytosis (ADCP) were higher at day 0 and day 3 in participants that cleared RNAemia compared to subjects with persistent RNAemia (Fig. 3C). Antibody-dependent neutrophil phagocytosis (ADNP) and neutralization levels (based on pseudovirus neutralization assay) showed a similar profile across the two groups at day 0 and day 7 but were significantly higher at day 3 in the clearance group (Fig. 3C). Overall, antibody functional profiles were compromised in individuals who exhibited prolonged RNAemia (Fig. 3D). In addition, the clearance group exhibited a more coordinated baseline antibody response at day 0, highlighted by higher levels of correlation across neutralization, ADCP, ADCD, and ADNP activity with several antibody features (IgG1 and IgA1 against all N/S/S1/S2/RBD, IgM against S/S1/S2/RBD, and FcγR binding; Fig. 3E and Fig. S3). Conversely, the persistence group exhibited a less coordinated functional humoral immune response, highlighting differences not only in univariate humoral immune trajectory differences across the groups, but also in the coordination and deployment of multiple antibody functions that may contribute collaboratively to control viral replication, dissemination, and drive viral clearance. While age, sex, hypertension, and other characteristics showed no impact on the kinetics or quality of humoral evolution, kidney disease and immunosuppression were modestly associated with humoral evolution (Fig. S4), pointing to immunomodulatory states that may perturb humoral evolution and thus compromise viral clearance. Furthermore, utilizing the nested mixed linear models, we evaluated the association between each antibody level among 50 measurements and persistence/clearance group information by explicitly controlling the effects of these comorbidities, including lung disease, kidney disease, diabetes, hypertension, and demographic information, including age and body mass index (BMI) (see Materials and Methods). We still observed that IgG2/3 against S1 and RBD, and IgG3 against S were enriched in the clearance group. Additionally, we observed that N-, S- and S1/RBD-specific responses all exhibited higher FcγR binding levels in the subjects with cleared infection than in those who experienced persistence. These results were consistent with univariate analysis without cofounder correction to suggest that clearance of RNAemia tracks with SARS-CoV-2-specific antibody evolution.

10.1128/mbio.01577-22.4 FIG S4 Association between baseline characteristics and antibody features. (A) −log10 *P* values derived from Mann-Whitney U tests to compare antibody features stratified by different baseline characteristics. *P* values were adjusted by the Benjamini-Hochberg method for multiple comparison. Only the adjusted *P* value of <0.05 was colored. (B) Mean difference in Z scores in antibody features stratified by different baseline characteristics. (C) Volcano plots of pairwise comparisons highlight differences between persistence and clearance by controlling for comorbidities including heart, lung, kidney disease, hypertension (HTN), and diabetes and demographic information including age and body mass index (BMI). The *x* axis represents the *t* value (normalized coefficient of the group variable in the full model, and the *y* axis denotes the *P* values by likelihood ratio test comparing the null model and full model). The null/full model represents the association between each individual measurement (response) and all collected clinical information with/without persistence and clearance group information (see Materials and Methods). The horizontal gray dashed line denotes where the *P* value equals 0.05, and the vertical gray dashed line denotes a manually selected threshold (*t* values = 2). Download FIG S4, TIF file, 1.5 MB. Copyright © 2022 Wang et al. 2022 Wang et al. https://creativecommons.org/licenses/by/4.0/ This content is distributed under the terms of the Creative Commons Attribution 4.0 International license.

### S2-, S-, and RBD-specific Fc-profiles distinguish clearance and persistence at day 3.

**FIG 4:**
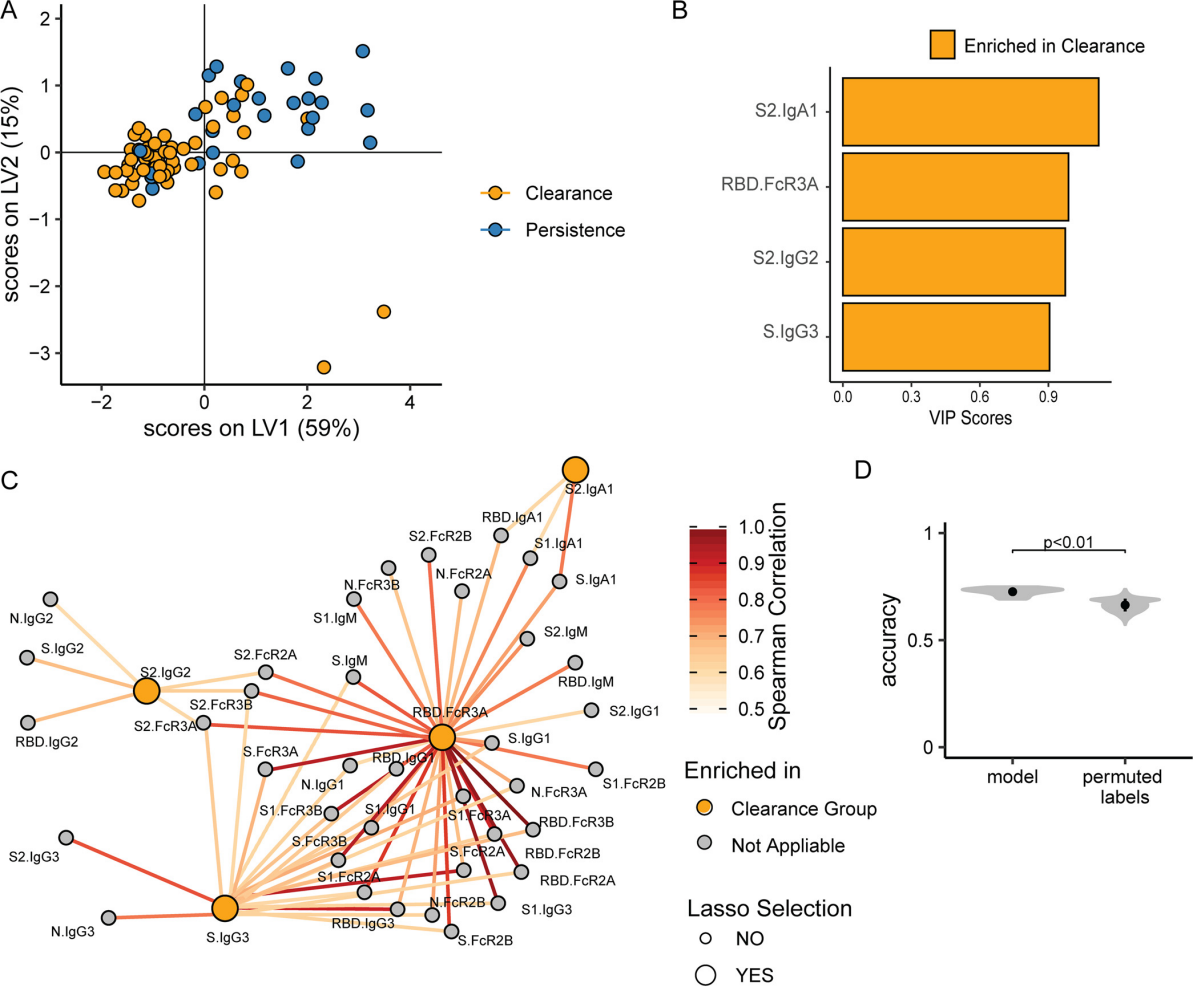
Multivariate analysis of antibody profiles across individuals that cleared or experienced persistent viremia. (A) Focusing on day 3, the PLSDA score plot demonstrates the degree of discrimination across individuals that cleared viremia or that experienced persistent viremia after LASSO feature down-selection. Each dot represents an individual; blue, persistence; orange, clearance. (B) The bar plot shows variable importance in projection (VIP) scores of the LASSO selected features. The magnitude of the bars indicates the importance of the feature in driving separation in the model. The color of the bar represents the group in which the feature is enriched. (C) The correlation network demonstrates the cocorrelated features (small nodes) that are significantly correlated with the model-selected features (large nodes). Edge color corresponds to the correlation strength. Here, only the significant Spearman correlation coefficients larger than 0.6 after Benjamini-Hochberg multiple testing correction are shown. (D) The violin plots show the distributions of repeated classification accuracy tests using the actual data and shuffled labels, illustrating the performance and robustness of the model. Black circles indicate the median accuracies with one standard deviation.

Given the multitude of humoral immune differences across subjects that cleared or exhibited persistence of RNAemia, we next aimed to define a minimal set of SARS-CoV-2 humoral signatures that account immunologically for systemic viral control and clearance. Least absolute shrinkage and selection operator (LASSO) feature selection (18) was performed first to collapse the multitude of correlated humoral features into a minimal set that maximally could account for variation in clearance of RNAemia. Separation between individuals that cleared the virus or exhibited persistence was then visualized using a partial least-squares discriminant analysis (PLSDA). Focusing on day 3, where the most significant univariate differences were observed, 74% of the variability across the groups could be explained by the first two latent variables (LVs) using as few as 4 selected features of the 50 total features that were captured for each plasma sample (Fig. 4A). The 4 features were S2-specific IgA1, S2-specific IgG2, S2-specific IgG3, and RBD-specific FcγR3A binding levels (Fig. 4B). Furthermore, given that the features selected during down-selection may represent additional cocorrelated humoral features that may provide additional mechanistic insights into the polyclonal mechanisms involved in elimination of RNAemia, we next built a cocorrelate network between selected features by LASSO and nonselected features. As shown in Fig. 4C, a single large cocorrelate network appeared, including the 4 model-selected features (large nodes), linked to additional highly correlated features (smaller nodes) pointing to a broader array of S1/S2/S-specific FcγR3A binding, RBD-specific FcγR2A/2B binding, and RBD-specific IgG1/IgG3, which likely collectively form a polyclonal response required for effective viral capture and clearance. In addition, the model performance was evaluated using 5-fold cross validation to test the significance of the model. The original model outperformed permuted controls (Fig. 4D). Additionally, we used the RNAemia value on day 7 as a continuous variable and further evaluated whether the similar humoral signatures were identified to associate with the continuous RNAemia. We performed a partial least-squares regression (PLS-R) analysis (Fig. S5) and observed that S2-specific IgA1 and S-specific IgG3 were also selected in this regression model. Additionally, through the correlation analysis, we observed that RBD-specific FcγR3A binding, selected in the classification model, was also highly correlated with S2-specific FcγR2A binding, which was selected in our regression model.

10.1128/mbio.01577-22.5 FIG S5 PLS-R regression model to associate the antibody profiles on day 3 and continuous values of viral load on day 7. (A) The relationship between continuous values of viral load on day 7 and our defined persistence versus clearance phenotype. (****, *P* < 0.00001). (B) the PLS-R score plot demonstrates the degree of regression between antibody profiles on day 3 and viral load measurements on day 7 across individuals after LASSO feature down-selection. Each dot represents an individual, and the color represents the continuous values of viral load on day 7. (C) The bar plot shows variable importance in projection (VIP) scores of the LASSO selected features. The magnitude of the bars indicates the importance of the feature in driving separation in the model. (D) The violin plots show the distributions of repeated regression R-square values using the actual data, and shuffled labels, illustrating the performance and robustness of the model. Black squares indicate the median accuracies with one standard deviation. (E) The correlation network demonstrates the cocorrelated features (small nodes) that are significantly correlated with the model-selected features (large nodes). Edge color corresponds to the correlation strength. Here, only the significant Spearman correlation coefficients larger than 0.6 after Benjamini-Hochberg multiple-testing correction are shown. Download FIG S5, TIF file, 1.2 MB. Copyright © 2022 Wang et al. 2022 Wang et al. https://creativecommons.org/licenses/by/4.0/ This content is distributed under the terms of the Creative Commons Attribution 4.0 International license.

### Antibody evolutionary kinetics distinguish clearance and persistence.

**FIG 5:**
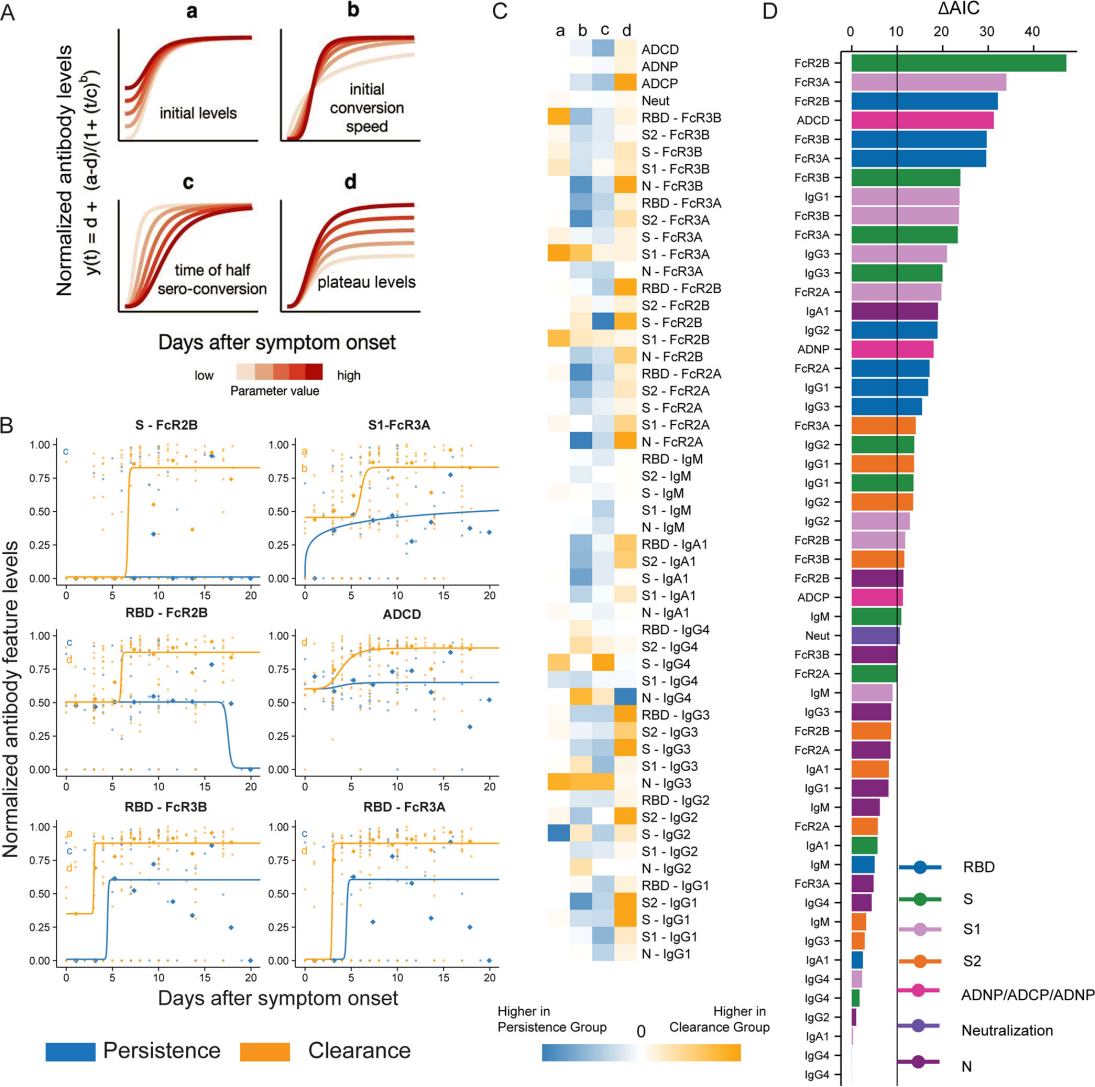
Modeling kinetics of antibody-mediated control of viremia. (A) The cartoon highlights the breakdown of the four-parameter logistic growth curve (a, initial levels; b, initial seroconversion speed; c, seroconversion time; d, endpoint levels) used to model the mechanism of antibody-mediated control of viremia. The influence of each parameter on the shape of the curve is shown for various parameter values. (B) The top six antibody features with highest ΔAIC values are shown as individual graphs depicting striking differences in kinetics across the two group-based days since symptom onset. Diamond-shaped dots indicate the binned median of measurements, and round dots indicate the measurement for each individual at certain time points. (C) The heatmap shows the AIC weight-averaged parameter differences between individuals that cleared viremia (yellow) or experienced persistent viremia (blue). The intensity of the color highlights the intensity of the enrichment of the feature in either group. Dots indicate individual patients, diamonds indicate the binned median, the curves indicate the optimal fitted models, and the colors indicate the groups. The parameters shown in the left corner are different for the displayed model and color-coded according to the group for which the parameter is higher. (D) The bar graph shows the ΔAIC of the four-parameter models. The bar heights are ranked based on features that explain trajectory differences best-to-worst- across the persistence and clearance groups. The vertical line (ΔAIC = 10) indicates the commonly used threshold for rejecting models.

Beyond differences in the humoral immune response at discrete time points, we next aimed to explore kinetic differences in the evolution of the humoral immune response to further mechanistically define the importance of distinct humoral features in clearance of RNAemia over time. Thus, SARS-CoV-2-specific responses were modeled based on time from symptom onset. Locally estimated scatterplot smoothing (LOESS) curves demonstrated significant differences in the evolutionary kinetics of all humoral features across the groups, marked by similar early responses, which plateaued lower in the persistence group, rapidly decaying to lower levels than those observed in the clearance group (Fig. S6). To quantify these differences, quadrimetric modeling was applied to these data (Fig. 5A and B, Fig. S7) to probe the specific differences in each humoral immune parameter based on initial levels at either the time of symptom onset (parameter a), in the initial rise or conversion speed (parameter b), in the time to half-seroconversion (parameter c), or in the ultimate final plateau level (parameter d) (Fig. 5A). The Akaike information criterion (AIC) was used to determine the best model for each antibody feature. Interestingly, initial levels were generally comparable across the groups, although higher levels of S-IgG2 were observed in the persistence group in contrast to the higher N-specific IgG3, S1-FcγR2b, S1-FcγR3A, and RBD-specific FcγR3B observed in the clearance group (Fig. 5C). Surprisingly, a faster initial rise and time to half-seroconversion was observed for most features in those who experienced viral persistence, with the exception of the initial features that were enriched in individuals with rapid RNAemia clearance (Fig. 5C). However, plateau magnitudes were largely elevated in individuals with rapid clearance of RNAemia, except for N-specific IgG4, a marker of a less functional humoral immune response in people with COVID-19. Among the antibody functions, ADCP, ADBP, and ADCD all plateaued at higher levels in the clearance group, while neutralization did not differ across the groups over time. These data collectively point to a highly specific early humoral immune response, marked by IgG3 and Fcγ-receptor binding characteristics that may be key for initial viral control, and the deficiencies in humoral immune responses linked to viral persistence (Fig. 5C).

10.1128/mbio.01577-22.6 FIG S6 Temporal evolution visualization of antibody features. Normalized antibody levels are shown over time per each measurement as days since symptom onset with persistence (bottom) and clearance (upper) groups. Each dot represents an individual measurement of an individual participant, and the curves show smoothed nonparameter regression (LOESS) models. The color of the line shows the antigen specificity. Download FIG S6, TIF file, 1.6 MB. Copyright © 2022 Wang et al. 2022 Wang et al. https://creativecommons.org/licenses/by/4.0/ This content is distributed under the terms of the Creative Commons Attribution 4.0 International license.

10.1128/mbio.01577-22.7 FIG S7 Temporal evolutionary curve of antibody features. For each antibody feature, the optimal model fitted by our four-parameter logistic regression model is shown for each group across each feature. Dots indicate individual patients, diamonds indicate the binned median, the curves indicate the optimal fitted models, and the colors indicate the groups. The parameters shown in the left corner are different for the displayed model and color-coded according to the group for which the parameter is higher (refer to Fig. 5). (a) Initial levels at the time of symptom presentation; (b) initial rise or conversion speed; (c) time to half-seroconversion; (d) the ultimate final plateau level. Download FIG S7, TIF file, 1.5 MB. Copyright © 2022 Wang et al. 2022 Wang et al. https://creativecommons.org/licenses/by/4.0/ This content is distributed under the terms of the Creative Commons Attribution 4.0 International license.

To finally define the antibody evolutionary profiles that differed most across the groups, changes in AIC values for each antibody feature were listed to determine features that showed the greatest variation between individuals that cleared RNAemia or experienced the persistence of RNAemia (Fig. 5D). Fcγ R binding levels dominated the top 10 most differential humoral immune features, marked by highly divergent S-, RBD-, and S1-specific titers and ADCD activity. Moreover, changes in longitudinal levels highlight temporal differences for each of the top 6 humoral features, pointing to a potential critical initial expansion of RBD-specific FcγR3B at the time of symptom onset in the viral clearance group, followed by rapid evolution of S- and RBD-specific Fcγ R2B binding antibodies, which stands in contrast to the low and waning levels found in the viral persistence group (Fig. 5B, Fig. S7). To evaluate the robustness of the model fit, we fit the model using 85% of the sample data randomly in three independent replicates and summarized the resulting most discriminatory features (Fig. S8A and B). The top 30 shared features were ordered based on their ΔAIC and the proportions were visualized using Venn diagrams (Fig. S8C). These data demonstrate the repeated emergence of shared discriminatory features across each analysis, further pointing to the importance of specific antibody Fc-binding and functional characteristics that may be key for therapeutic activity of SARS-CoV-2 antibodies in the setting of an evolving humoral immune response in COVID-19.

10.1128/mbio.01577-22.8 FIG S8 Four-parameter logistic regression model evaluation. The model performance was evaluated in three runs using randomly selected 85% sample sets. (A) The bar graphs show the ΔAIC of the four-parameter models in three runs. (B) The heatmaps show the AIC weighted-average parameter difference between individuals that cleared RNA (yellow) or experience persistent RNAemia (blue). (C) The Venn diagram depicts the shared features among the top 30 features across the original experiments and three runs with partial datasets. Download FIG S8, TIF file, 2.4 MB. Copyright © 2022 Wang et al. 2022 Wang et al. https://creativecommons.org/licenses/by/4.0/ This content is distributed under the terms of the Creative Commons Attribution 4.0 International license.

## DISCUSSION

In the wake of the completion and early termination of several large convalescent plasma and monoclonal therapeutic trials (8, 19–21), whether and how antibodies contribute to viral control and clearance in the setting of severe disease remains unclear. Monoclonal therapeutics have been shown to confer benefit prior to the evolution of the natural humoral immune response (11). However, naturally evolving antibody functions have been linked to survival and clearance across several studies (9, 12). Thus, in this study, we sought to define the specific antibody functional profiles that track with antiviral control and the resolution of COVID-19, focusing on the dynamics of SARS-CoV-2 RNAemia, known to be strongly associated with clinical outcomes. Here, we observed a strong linkage between RNAemia and particular SARS-CoV-2-specific antibody Fc-characteristics, pointing to the early importance of IgG3, S1/RBD Fcγ-receptor binding activities, and evolution of broad FcγR and opsonophagocytic functions in individuals that rapidly clear SARS-CoV-2 RNAemia. These data highlight the critical role of particular Fc-receptor functions as key functional determinants that may be essential for clearance of SARS-CoV-2 RNAemia, in the setting of a massively proliferating antiviral immune response, which may be key to resolution of severe disease.

We and others have previously reported in cross-sectional studies that detectable SARS-CoV-2 RNAemia and viral dissemination is associated with increased risk of severe COVID-19 and mortality (1, 3, 22, 23), which appears to be mediated by increased systemic inflammation, endothelial and tissue damage, and perturbation of the coagulation cascade (1, 24). While prolonged RNAemia has been described in immunosuppressed individuals (4, 25), its role in predicting disease severity in the general population has remained unclear. In this study, we further demonstrated that failure of prompt SARS-CoV-2 RNAemia clearance is an independent predictor of maximal COVID-19 severity and risk of mortality, even after adjusting for baseline levels of RNAemia and other potential confounders. These findings provide important context on the importance of identifying immune factors that lead to rapid clearance of plasma RNAemia. Our findings are consistent with cohort studies from other medical centers that suggest that longer duration of RNAemia and lower RBD-specific IgG responses are related to worse COVID-19 outcomes (23, 26, 27), extending these observations to the specific antibody characteristics that may underly antibody-mediated antiviral clearing activities.

Previous studies have pointed to early antibody functional evolution as a correlate of natural resolution of COVID-19 (9, 10, 12). However, whether specific humoral changes were linked to systemic control of RNAemia, and thus to control of viral dissemination and broader disease, was unclear. In our current study, we revealed that early development of certain antibody features (S-IgG3, S-Fcγ R2B binding, and RBD-FcγR3B binding) are associated with absence of RNAemia in the early course of disease. However, in people who had detectable viremia at the time of emergency department presentation, rapid and robust evolution of broad Fc-receptor binding profiles and functions are tightly linked to the kinetics of viremia clearance. These data are consistent with earlier reports suggesting that the timing and maintenance of antibody functions, rather than cross-sectional antibody levels and neutralization titers, are more tightly associated with COVID-19 outcomes (12). Similarly, in a New Haven cohort, 50% of discharged COVID-19 participants mounted neutralizing antibodies in the first 7 to 9 days of symptom onset, compared to slower kinetics (~14 days) in those who ultimately passed away (9). Delayed humoral immune evolution may be explained by defective Bcl-6-expressing T follicular helper cell evolution in severe/fatal COVID-19 leading to delayed and defective humoral immune evolution (28). Thus, collectively, our data demonstrate that early and rapid antibody development, especially certain SARS-CoV-2 spike-specific IgG FcγR binding properties, may contribute centrally to the prevention of viral dissemination and, more importantly, clearance of viral dissemination once RNAemia develops.

While convalescent therapy and monoclonal therapeutics have shown efficacy in individuals with low-level baseline SARS-CoV-2 antibody titers during early presentation (29), these therapeutics have shown more limited impact following the evolution of the humoral immune response (7, 8, 11, 21). This modest effect during progressive disease may relate to the necessity for antibody therapeutics to compete for immune complex occupancy with naturally evolving humoral immune responses, which may become progressively more difficult as the humoral immune response evolves. The data presented here, however, may provide an additional explanation for the modest performance of these antiviral therapies, related to the importance of antibodies to bind more effectively to Fc-receptors (30) and aggressively draw on innate immune function early in disease to reprogram the activity of evolving immune complexes. In a mouse model, Suryadevara and colleagues demonstrated that FcγR binding is required for the therapeutic activity of 2 monoclonal therapeutics (COV2-2676 and COV2-2489) following SARS-CoV-2 infection (31). Similarly, Yamin and colleagues demonstrated that optimizing FcγR binding via monoclonal antibody (MAb) engineering was associated with better protection against SARS-CoV-2 (32). These findings were further confirmed in a live-imaging mouse model demonstrating that FcγR-effector cell interaction was crucial in preventing severe disease by dampening inflammatory responses (33). Along these lines, recent correlate analysis from the CONCOR-1 convalescent plasma study pointed to the enrichment of elevated antibody-dependent cellular cytotoxicity (ADCC) in survivors compared to deceased individuals (21). Furthermore, the competition of monoclonal antibodies with the more multifaceted host humoral immune responses could help explain the link between signals of treatment antagonism with worse outcomes in hospitalized patients who already have a developed humoral immune response (7, 8). Thus, emerging next-generation monoclonal antibodies have begun to explore the potential addition of Fc-enhanced monoclonal drugs, which may contribute functionally to the evolving humoral immune response, shifting and potentiating antiviral control and clearance in a way that has not been explored by current Fc-silent or simple IgG1 backbones to date.

Because the participants in this study presented to the ED with various durations of symptoms, which were subjectively reported, we aligned all the samples from different individuals based on day of symptom onset and performed quantitative kinetics modeling to ensure that our findings still hold true. This analysis provided critical assurances that differences in kinetics were not attributable to simple differences in time from infection. Peripheral blood mononuclear cells (PBMC) were also collected in this cohort, but SARS-CoV-2-specific T cell analyses have not been performed in this cohort. Additional large collections are under way to begin to probe the complementary role of antibody effector functions and T cells in control of RNAemia (34, 35). However, despite these limitations, the study presented here, linking SARS-CoV-2 RNAemia dynamics and virus-specific antibody evolution, points to critical humoral functions that may play a key role in preventing dissemination and contributing to control and clearance of the disease. These results provide insights for next-generation monoclonal therapeutic design, especially in the face of new variants, including Omicron (36, 37).

## MATERIALS AND METHODS

### Participants.

Participant enrollment was described in our prior studies (1, 13). Participants were enrolled in the emergency department (ED) from Massachusetts General Hospital, Boston, MA, from 24 March 2020 to 30 April 2020 during the first peak of the COVID-19 surge, with an institutional review board (IRB)-approved waiver of informed consent. Inclusion criteria for this cohort included COVID-19-related symptoms, adults, and nucleic acid test confirmation of SARS-CoV-2 infection. Clinical course was followed to 28 days postenrollment or until hospital discharge if that occurred after 28 days.

### Plasma SARS-CoV-2 RNAemia quantification.

Plasma SARS-CoV-2 viral load measurement was reported in our previous study (1, 13). Briefly, RNA was extracted from 300 μL of RPMI 1640-diluted EDTA-preserved plasma sample (RPMI 1640:plasma 2:1 dilution) (1, 13) using a TRIzol‐based method (Thermo Fisher Scientific, Waltham, MA). SARS-CoV-2 viral load was quantified using the U.S. CDC2019-nCoV_N1 primers and probe set (3). Viral load reverse transcriptase PCR (RT-PCR) assays were performed in triplicate. The lower limit of SARS-CoV-2 N gene quantification was 100 copies/mL. Samples with a positive signal but viral load of <100 copies/mL were denoted detectable but unquantifiable and were given a value of 10 copies/mL (1 log copy/mL).

### RNAemia clearance.

The majority of participants only had blood samples from days 0, 3, and 7 of hospitalization available. Thus, it is difficult to categorize their RNAemia clearance based on a snapshot of day 7 viral load with limited duration of follow-up, given that participants with different baseline RNAemia levels may exhibit different viral load dynamics between day 0 and day 7 (Fig. 2A). We define RNAemia clearance based on RNAemia level at day 0:

• For participants with day 0 viral load of ≥3 log copies/ml: day 7 viral load at least unquantifiable (<2 log copies/mL) or day 3 viral load at least unquantifiable (<2 log copies/ml if day 7 viral load not available).
• For participants with day 0 viral load of <3 log copies/mL: day 7 viral load undetectable or day 3 viral load at least unquantifiable (<2 log copies/mL if day 7 viral load not available).

Those who did not satisfy these criteria were categorized in the RNAemia persistence group.

In addition, there were four participants with baseline day 0 viral load unquantifiably detectable but that only had day 3 follow-up sample available. All four participants had day 3 viral load unquantifiably detectable. These four participants were grouped in the persistence group.

### Luminex-based antibody levels and FcγR binding assays.

Antigen-specific antibody subclasses and FcγR binding levels were measured using a 384-well-based customized multiplexed Luminex assay as previously described (14). In this study, the following antigen-specific antibody levels were measured using this high-throughput platform SARS-CoV-2 nucleocapsid (N) protein (Aalto Bio Reagents), SARS-CoV-2 spike protein (S) (kindly provided by Eric Fischer, Dana Farber), S1 (Sino Biological; 40591-V08B1), S2 (Sino Biological; 40590-V08B), and SARS-CoV-2 RBD (kindly provided by Aaron Schmidt, Ragon Institute). Briefly, antigens were covalently bound to fluorescent carboxyl-modified microspheres (Luminex) by *N*-hydroxysuccinimide (NHS)-ester linkages using 1-Ethyl-3-(3-dimethylaminopropyl) carbodiimide (EDC) and sulfo-NHS (Thermo Scientific). Antigen-coupled beads were then washed and blocked before adding plasma samples at an appropriate sample dilution (1:500 for IgG1, 1:1,000 for all Fc- receptors, and 1:100 for all other isotype/subclass readouts). The sample then underwent an overnight incubation at 4°C with shaking at 700 rpm, followed by washing with an automated plate washer (Tecan) with 0.1% bovine serum albumin (BSA) and 0.02% Tween 20. Antigen-specific antibody titers were detected using a phycoerythrin (PE)-coupled detection antibody for each subclass and isotype (IgG1, IgG2, IgG3, IgA1, and IgM; Southern Biotech), and Fc-receptors were fluorescently labeled with PE before addition to immune complexes (FcγR-2A, -2B, -3A, and -3B; Duke Protein Production facility). Plasma samples were acquired via flow cytometry, using an iQue (IntelliCyt) platform and S-LAB robot (PAA). Analysis was done using ForeCyt software by gating on fluorescent bead regions, and PE median fluorescent intensity (MFI) was reported as the readout for antigen-specific antibody titers and FcγR binding levels.

### Effector function assays.

Bead-based assays were used to quantify antibody-dependent cellular phagocytosis (ADCP), antibody-dependent neutrophil phagocytosis (ADNP), and antibody-dependent complement deposition (ADCD) as previously described (12, 38). Briefly, yellow (ADNP and ADCP) as well as red (ADCD) fluorescent neutravidin beads (Thermo Fisher) were coupled to biotinylated SARS-CoV-2 RBD, N, and S antigens and incubated with diluted plasma (ADCP and ADNP 1:100, ADCD 1:10) to allow immune complex formation for 2 h at 37°C.

For ADCP, THP-1 cells (ATCC) were added to the immune complexes at 1.25 × 10^5^cells/mL and incubated for 16 h at 37°C. Events were gated on singlets and bead-positive cells.

For ADNP, HL-60 cells were differentiated into CD11-expressing neutrophils and then to immune complexed yellow beads and incubated for 16 h at 37°C. Afterward, neutrophils were stained with an anti-CD11 BV605 detection antibody (BioLegend) and fixed with 4% paraformaldehyde (Alfa Aesar). Events were gated on CD11+bead+ neutrophils.

For ADCD, lyophilized guinea pig complement (Cedarlane) was reconstituted and diluted in gelatin veronal buffer with calcium and magnesium (GBV++) (Boston BioProducts). Subsequently, C3 was detected with an anti-C3 fluorescein-conjugated goat IgG fraction detection antibody (MP Bio). ADCD was reported as the median of C3 deposition.

All assays were acquired via flow cytometry with an iQue (IntelliCyt) platform and an S-LAB robot (PAA). Phagocytosis scores were calculated for ADCP and ADNP as (percentage of bead-positive cells) × (MFI of bead-positive cells) divided by 10,000.

### Neutralization.

The pseudovirus-based neutralization assay was reported in our prior study (13). Briefly, lentivirus vector was constructed using PCR amplification (Q5 high-fidelity 2× master mix; New England Biolabs) from pUC57-nCoV-S (gift of Jonathan Abraham) and further fused to HIV-1 gp41 to obtain pCMV-SARS2ΔC-gp41. 293T cells were transfected with 1 μg psPAX, 1.6 μg pTRIP-SFFV-EGFP-NLS (Addgene), and 0.4 μg pCMV-SARS2ΔC-gp41 using TransIT-293 transfection reagent (Mirus Bio). After overnight incubation, the medium was changed. SARS-CoV-2 S pseudotyped lentiviral (Wuhan-Hu-1 strain) particles were collected 30 to 34 h post-medium exchange and filtered using a 0.45-μm syringe filter. One day before the neutralization experiment, 293T ACE2/TMPRSS2 cells were seeded at 5 × 10^3^ cells in 100 μL per well in 96-well plates. On the day of lentiviral harvest, 100 μL SARS-CoV-2 S pseudotyped lentivirus was incubated with 50 μL of plasma diluted in medium to a final concentration of 1:100. Medium was then removed from the 293T ACE2/TMPRSS2 cells and replaced with 150 μL of the mix of plasma and pseudotyped lentivirus. Wells in the outermost rows of the 96-well plate were excluded from the assay. After overnight incubation, medium was changed to 100 μL of fresh medium. Cells were harvested 40 to 44 h postinfection with TrypLE (Thermo Fisher), washed in medium, and fixed in fluorescence-activated cell sorter (FACS) buffer containing 1% paraformaldehyde (PFA; Electron Microscopy Sciences). The percentage of green fluorescent protein (GFP) was quantified on a CytoFLEX LX instrument (Beckman Coulter), and data were analyzed with FlowJo. The events recorded by the flow cytometer do not suggest cell loss due to detachment. Neutralization rate was defined as 1 − (GFP% pseudovirus + plasma/GFP% pseudovirus alone).

### Statistics.

Continuous variables were summarized using medians and interquartile ranges (IQRs). For clinical variables, we used the Mann-Whitney test to compare continuous variables from two different categorical groups and Dunn’s test with the Benjamini-Hochberg *post hoc* test for three or more groups. Categorical variables were evaluated using the χ^2^ test or Fisher’s exact test. We used Spearman’s rank correlation coefficient to evaluate correlation between different continuous variables. To evaluate the association of SARS-CoV-2 RNAemia clearance and clinical outcomes, we used logistic regression analyses to calculate odds ratios (OR) and 95% confidence intervals (CI). Clinical data analyses and logistic regression were performed on Stata (version 13.1), and figures were generated by Stata and GraphPad Prism (version 9.1). R (version 4.0.0) and Python (version 3.6.8) were used to analyze antibody data as described below.

### Temporal analysis.

The full methods were described in our recent study (12). Briefly, all antibody features were subtracted by phosphate-buffered saline (PBS) control values and then log_10_ transformed. After that, the values were normalized to a (0, 1) scale such that the minimal value equals 0 and the maximal value equals 1. First, a nonparametric regression model was used to obtain a smoothed line using the R function loess (span = 0.7). Of note, the late rise and fall of some curves is attributable to a limited number of late time points and not due to a true elevation in antibody levels.

Four-parameter logistic growth curve regression was described in our recent study (12).

$$y\left(t\right)=d+\frac{a-d}{\left(1+{\left(\frac{t}{c}\right)}^{b}\right)}$$

In this equation, the four parameters denote the following features: *a*, the initial antibody levels upon symptom onset; *b*, the initial seroconversion speed; *c*, the time of 50% seroconversion; and *d*, the plateau end levels. In order to compare antibody dynamics from clearance and persistence groups, models were built for these two groups simultaneously, allowing for combinations of parameters to differ between the groups, leading to 2^4^ = 16 models. For each feature, each of the 16 models was fitted to the data using maximum likelihood estimation (Laplacian likelihood function to handle outliers), treating each measurement as an independent data point and assuming that differences in measurements arose due to measurement noise. We used the Akaike Information Criterion (AIC) to decide which particular differences were most distinct across two groups (39). The model with the lowest AIC value was then chosen to be the best model.

### Association analysis between measured antibody levels and persistence/clearance by controlling potential cofounders.

Utilizing a nested mixed linear model (null and full model) with/without comorbidities and physical information, we accessed the significance of the association between antibody-related measurements and defined persistence/clearance group information. Using samples caught within 21 days since symptom onset, we fit 2 linear mixed models and estimated the improvement of model performance using a likelihood ratio test for each measurement. Then, we evaluated the significance of the association as the improvement of model performance (*P* ≤ 0.05) and the coefficient of the persistence/clearance group information. The details of the nested linear mixed models are shown as follows:

Null model:

$$\mathrm{antibody}\;\mathrm{measuremen}t_{\mathrm{ij}}\sim 1+\mathrm{Ag}e_{i}+\mathrm{BM}I_{i}+\mathrm{Hear}t_{i}+\mathrm{Lun}g_{i}+\mathrm{Kidne}y_{i}+\mathrm{Diabete}s_{i}+\mathrm{HT}N_{i}+\mathrm{ImmunoRepressio}n_{i}+\mathrm{SymOnse}t_{\mathrm{ij}}+(1|\mathrm{Pat}.ID_{i})$$

Full model:

$$\mathrm{antibody}\;\mathrm{measuremen}t_{\mathrm{ij}}\sim 1+\mathrm{Persistance}{?}_{i}+\mathrm{Ag}e_{i}+\mathrm{BM}I_{i}+\mathrm{Hear}t_{i}+\mathrm{Lun}g_{i}+\mathrm{Kidne}y_{i}+\mathrm{Diabete}s_{i}+\mathrm{HT}N_{i}+\mathrm{ImmunoRepressio}n_{i}+\mathrm{SymOnse}t_{\mathrm{ij}}+(1|\mathrm{Pat}.ID_{i})$$

Likelihood ratio test:

$$\mathrm{LRT}=-2^{\text{*}}\ln\left(\frac{\mathrm{MLE}\;\mathrm{in}\;\mathrm{full}\;\mathrm{model}\;}{\mathrm{MLT}\;\mathrm{in}\;\mathrm{null}\;\mathrm{model}}\right)\sim \lambda^{2}$$

Here, demographic information including age and body mass index (BMI) and the binary status of historical diseases including heart disease, lung disease, kidney disease, diabetes, hypertension (HTN), and immunosuppression status were included into the models as fixed potential confounders/effects. Additionally, the day of symptom onset (SymOnset) was incorporated into the model, and the different initial values (intercept) of measurement among different patients were included as a random effect.

The R package lme4 was used to fit two nested models to each measurement. The *P* value from the likelihood ratio test and the *t* value (normalized coefficient) of the persistence/clearance group information in the full model were visualized as a volcano plot using the ggplot function in the R package ggplot2.

### Partial least-squares discriminant analysis (PLSDA)/partial least-squares regression analysis (PLS-R).

First, we applied the least absolute shrinkage and selection operator (LASSO) feature selection algorithm to extract significant features. We ran LASSO 10 times on the whole data set and identified the set of features in more than 60% of the repetitions, which were implemented in the function select_lasso in the systemseRology R package (version 1.0). The PLSDA model was built using the extracted features. Model performance was evaluated by 5-fold cross-validation, and negative-control models were constructed from permuted data with 100 iterations. For PLS-DA, we used the opls function in the ropls R package (version 1.22.0) for classification and functions in the systemseRology R package for the purpose of visualization. Model training details were reported in our recent system serology study on SARS-CoV-2 (40). A partial least-squares discriminant analysis (PLS) classifier was then trained using the fold-specific selected features to predict the test set. Multiple iterations of fold-specific feature selections were performed to obtain a single model. This process was repeated over 20 replicates, and convergent correlates were observed. The performance and robustness of the model were contrasted with those of negative-control models from permuted data with 100 iterations of 5-fold cross-validation to generate classification accuracy. In detail, the permuted control was generated by shuffling labels randomly for each time. Robustness was defined as the effect size of distributions and the exact *P* values of the tail probabilities of the actual distribution with the control distribution. For the regression analysis, the same procedure was applied to the data set, and PLS-R in the ropls R package function was used to evaluate the association between selected features and the continuous response (RNAemia values).

### Correlation networks.

We constructed correlation networks to visualize the additional humoral immune features that were significantly linked to the selected minimal antibody features. In brief, antibody features that were significantly correlated with a Holms-Bonferroni correction to the final selected PLS model selected features were defined as cocorrelates. Significant Spearman correlations above a threshold of |r| ≥ 0.6 were visualized within the networks. For implementation, Spearman correlation coefficients were calculated using the rcorr function in the Hmisc package (version 4.4.2), and the *P* values were corrected by Benjamini-Hochberg correction in the stats package (version 4.0.3). Finally, the networks were visualized using the ggraph and igraph packages with proper manual adjustment.
