# Supplementary figures and images for: The Kinetics of SARS-CoV-2 Antibody Development Is Associated with Clearance of RNAemia
Source: mBio. 2022 Jun 28;13(4):e01577-22. doi: 10.1128/mbio.01577-22 (PMC9426503; doi:10.1128/mbio.01577-22)

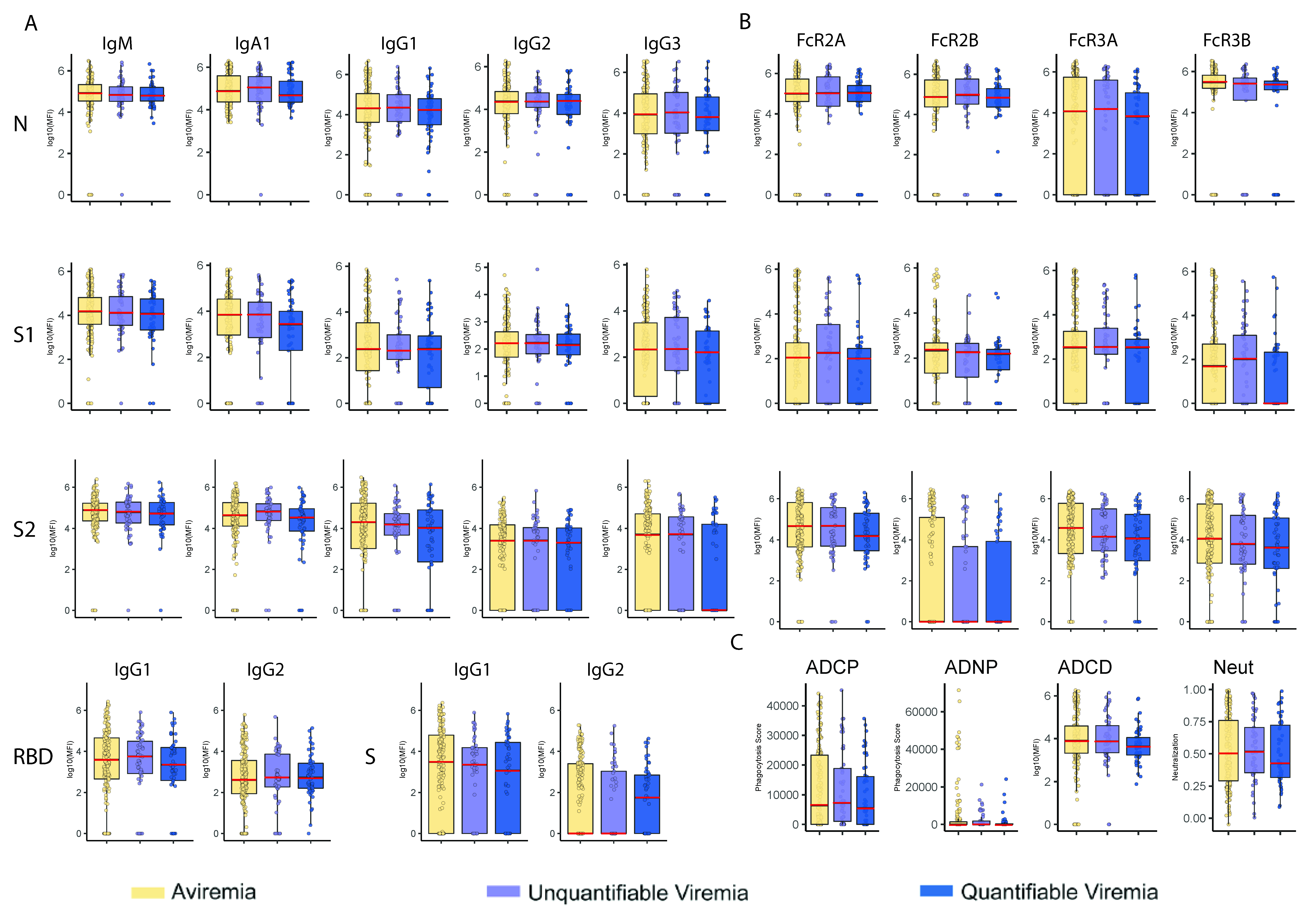

Supplement: FIG S1 [file mbio.01577-22-s0001.tif]

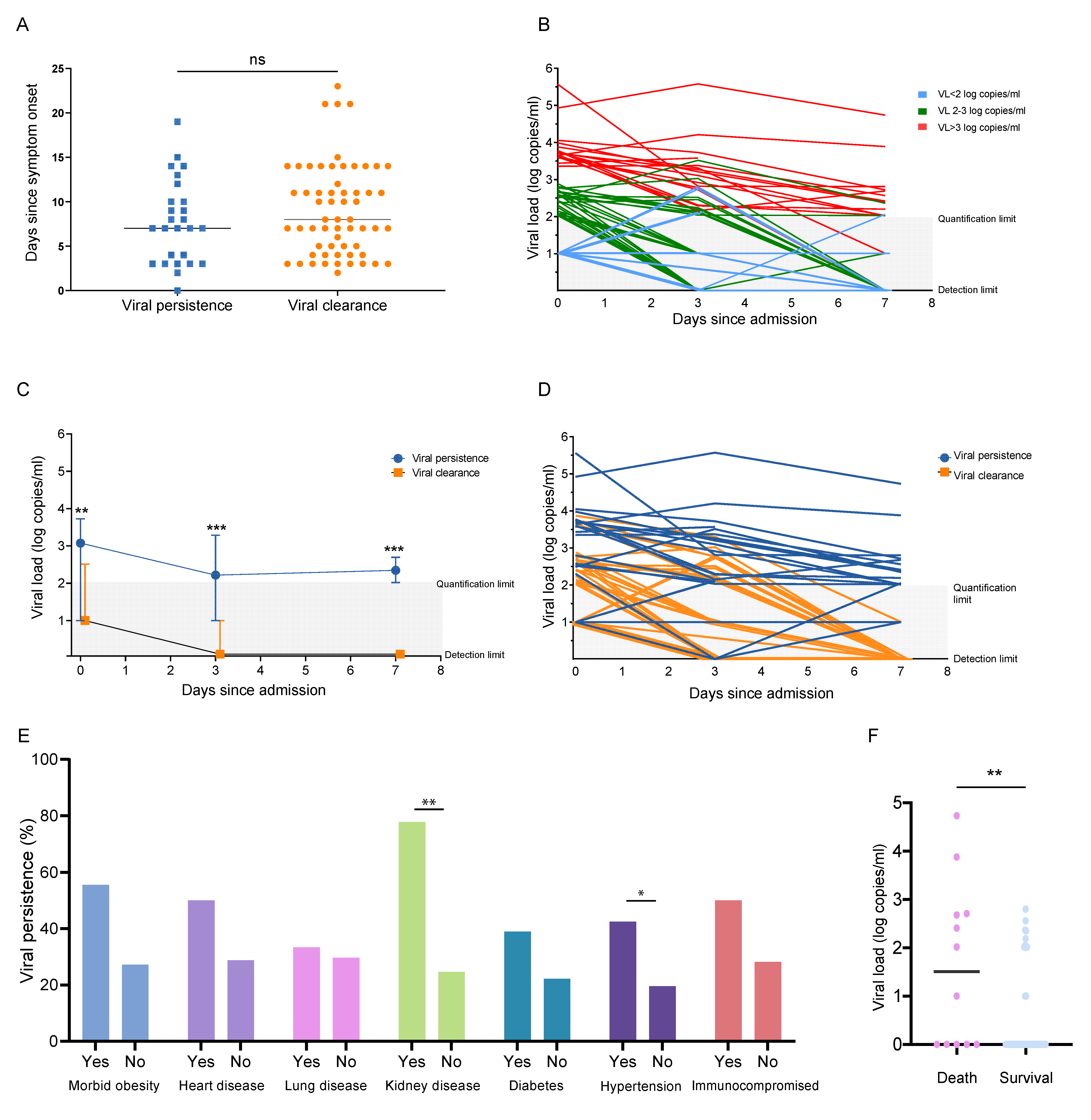

Supplement: FIG S2 [file mbio.01577-22-s0002.tif]

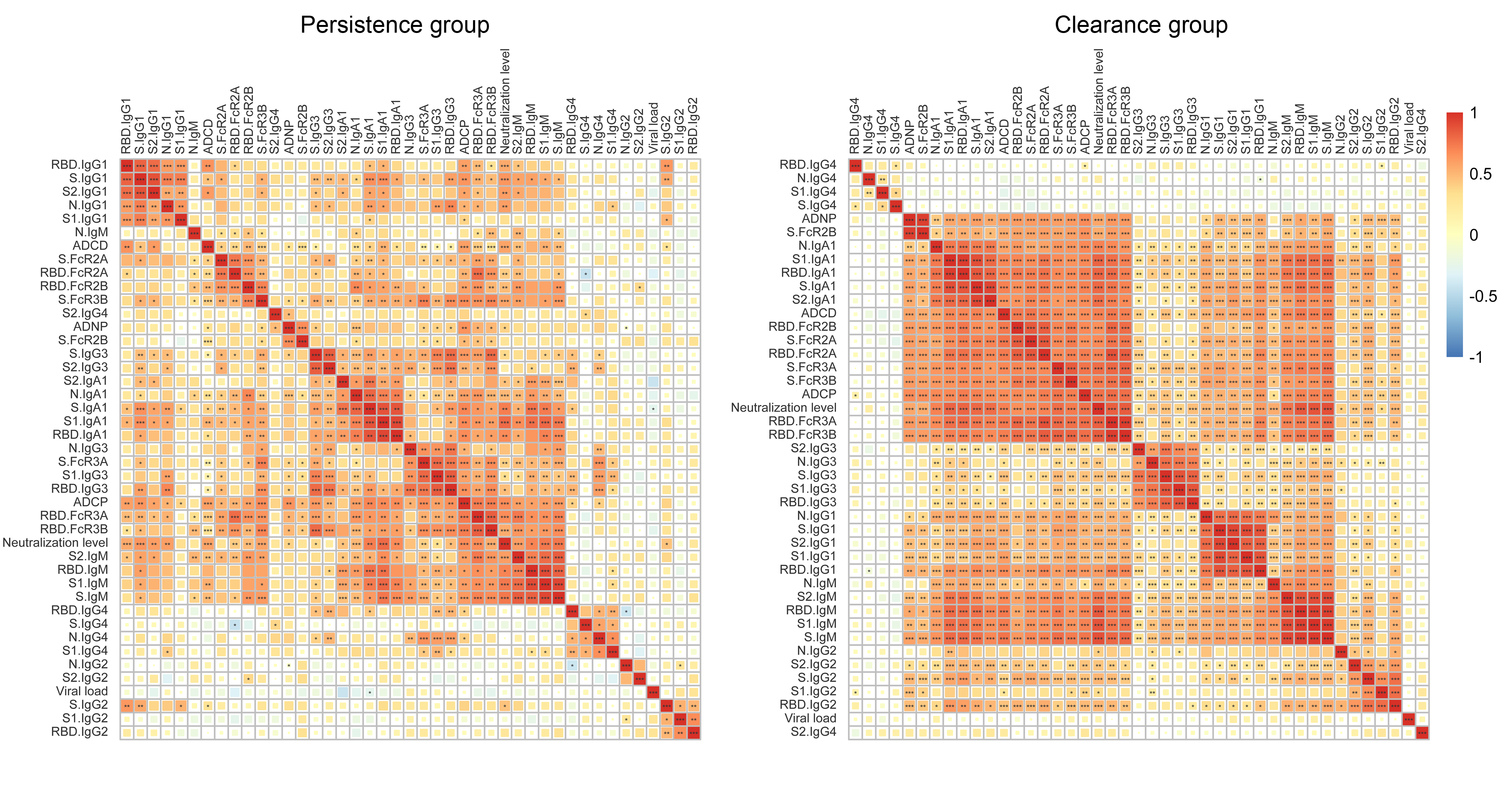

Supplement: FIG S3 [file mbio.01577-22-s0003.tif]

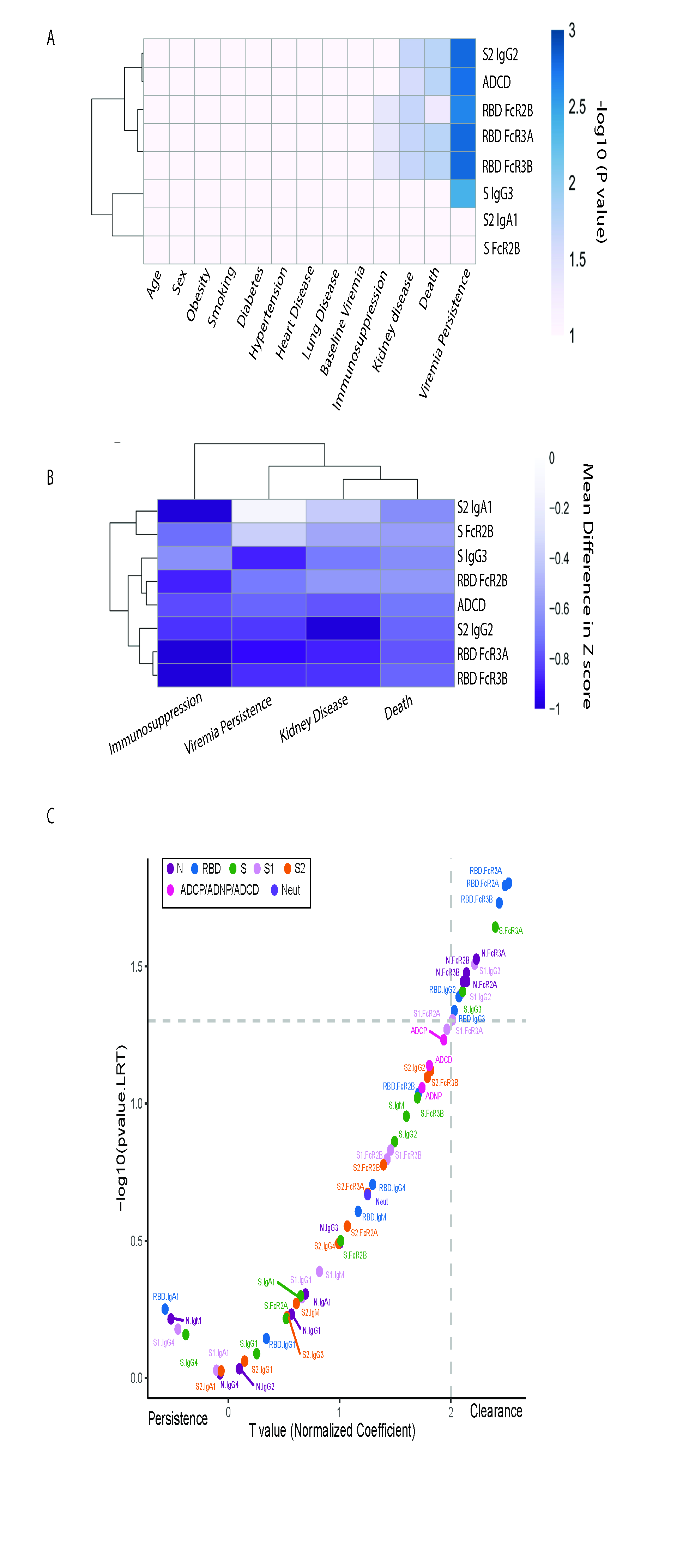

Supplement: FIG S4 [file mbio.01577-22-s0004.tif]

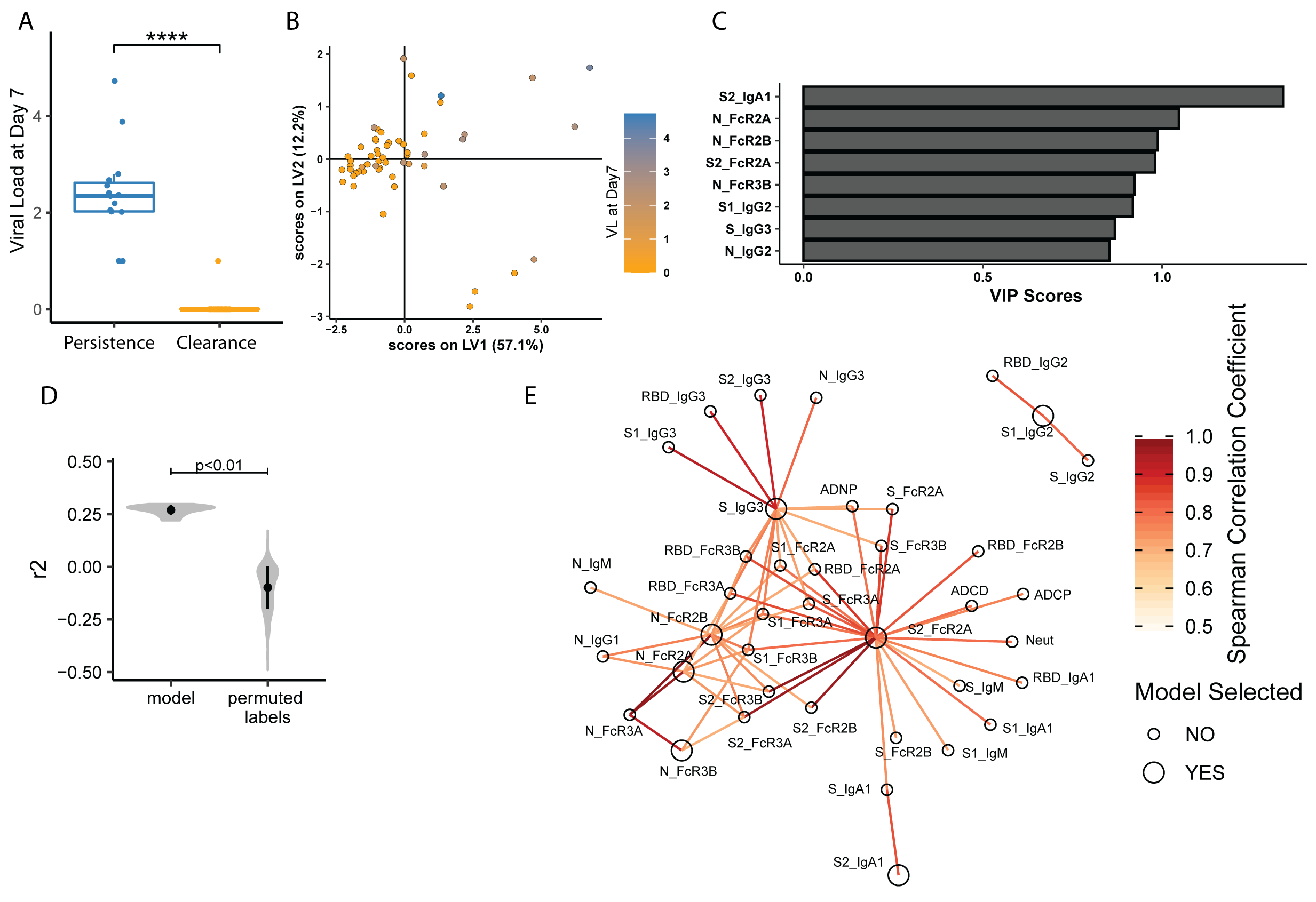

Supplement: FIG S5 [file mbio.01577-22-s0005.tif]

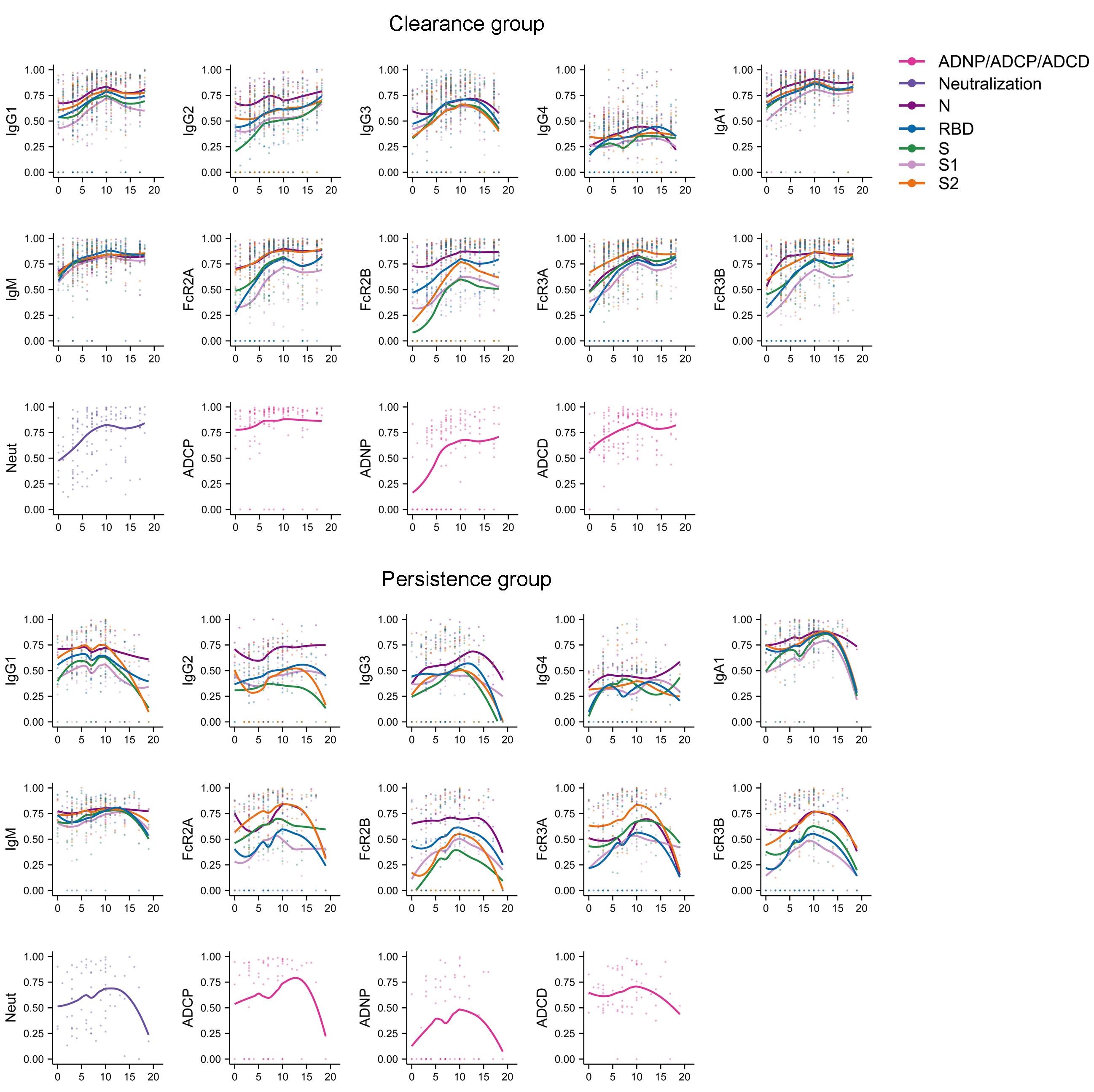

Supplement: FIG S6 [file mbio.01577-22-s0006.tif]
